# Role and interaction of bacterial sphingolipids in human health

**DOI:** 10.3389/fmicb.2023.1289819

**Published:** 2023-10-23

**Authors:** Xiaoye Bai, Ru Ya, Xiaoyu Tang, Mingwei Cai

**Affiliations:** ^1^School of Medicine, Sun Yat-sen University, Shenzhen, China; ^2^Shenzhen Bay Laboratory, Institute of Chemical Biology, Shenzhen, China; ^3^Inner Mongolia Academy of Science and Technology, Hohhot, China

**Keywords:** sphingolipids, bacteria, gut microbiome, human health, interaction

## Abstract

Sphingolipids, present in both higher animals and prokaryotes, involving in cell differentiation, pathogenesis and apoptosis in human physiological health. With increasing attention on the gut microbiome and its impact on wellbeing, there is a renewed focus on exploring bacterial sphingolipids. This review aims to consolidate the current understanding of bacterial sphingolipids and their impact on host health. Compared to mammalian sphingolipids, bacterial sphingolipids are characterized by odd chain lengths due to the presence of branched alkyl chains. Additionally, intestinal microbial sphingolipids can migrate from the gut to various host organs, affecting the immune system and metabolism. Furthermore, the intricate interplay between dietary sphingolipids and the gut microbiota is explored, shedding light on their complex relationship. Despite limited knowledge in this area, this review aims to raise awareness about the importance of bacterial sphingolipids and further our understanding of more uncharacterized bacterial sphingolipids and their significant role in maintaining host health.

## 1. Introduction

Sphingolipids are a group of lipids characterized by long-chain bases serving as the backbone, along with an amine group and two or three hydroxy groups at the structural end (Merrill, 2011). Sphingolipids, in the beginning, were thought to only exist in higher animals. However, subsequent research discovered sphingolipids in flagellates, rumen bacteria, protozoa, and specific Bacteroidaceae species such as *Bacteroides (B.) melaninogenicus* and *B. thetaiotaomicron*. These species are characterized by branched-chain sphingolipids (Stoffel et al., 1975; Olsen and Jantzen, 2001; Johnson et al., 2020; Le et al., 2022). As the gut microbiome and human health garner more attention, bacterial sphingolipids, particularly intestinal microbial sphingolipids, have gained interest due to their potential connections to gut microbiome and host health. Studies have shown that bacterial sphingolipids can be absorbed and detected in various organs throughout the body (Fukami et al., 2010; Johnson et al., 2020; Le et al., 2022). Additionally, sphingolipids produced by prominent *Bacteroidetes* in the gut have been found to impact host lipid metabolism and liver function (Johnson et al., 2020). Moreover, intestinal microbial sphingolipids play a significant role in maintaining host immune homeostasis (An et al., 2014; Brown et al., 2019). Despite the work of laboratories like Elizabeth Johnson’s Lab at Cornell University, there have been relatively fewer reports on bacterial sphingolipids compared to mammal sphingolipids.

There is evidence suggesting that many microorganisms, microbial toxins, and viruses bind to cells through sphingolipids (Vesper et al., 1999) and bacteria can translocate to host organs (Brown et al., 2019; Johnson et al., 2020). Several intriguing clinical phenomena have emerged, indicating the potential prospects of studying intestinal microbial sphingolipids in clinical research. For instance, it has been reported that dietary gangliosides may play a crucial role in modifying the intestinal microflora and promoting the development of intestinal immunity in neonates, thereby preventing infections during early infancy (Rueda, 2007). Another study examined the gut microbiome and neurodevelopment in infants from a general population birth cohort at two critical periods during infancy. The findings revealed that infants with a dominance of *Bacteroidetes* displayed enrichment in multiple metabolic functions, including sphingolipid metabolism and glycosphingolipid biosynthesis. This group of infants also achieved higher scores in cognitive, language, and motor development at the age of 2 years old (Tamana et al., 2021). These fragmented pieces of information emphasize the importance of investigating bacterial sphingolipids, including their unidentified compounds and structures, their impact on host physiology, and their role in connecting the gut microbiome with host health. Understanding how the daily diet influences the gut microbiome and its sphingolipids, as well as comprehending the mechanisms by which intestinal microbial activity affects host health, are important areas that require extensive research. It is clear that addressing these complex questions will require considerable effort and time.

This review provides a comprehensive overview of current knowledge regarding bacterial sphingolipids and their impact on host health. We will begin by introducing sphingolipids in general and highlight the structural differences between bacterial sphingolipids and those found in mammals. Additionally, we examine the influence of bacterial sphingolipids on the host’s immune system and metabolites. Furthermore, we will explore the intricate relationship between dietary sphingolipids and the gut microbiota. Despite limited knowledge in this area, we aim to contribute to the understanding of bacterial sphingolipids. Our ultimate objective is to raise awareness and further investigate the importance of bacterial sphingolipids, particularly intestinal microbial sphingolipids, and provide potentials to develop targeted therapeutics for uncharacterized sphingolipids. Additionally, we aim to elucidate the underlying mechanisms by which these sphingolipids interact with the gut and influence host health.

## 2. An overview of sphingolipid structure and categories

### 2.1. Definition and categories of sphingolipids

**FIGURE 1:**
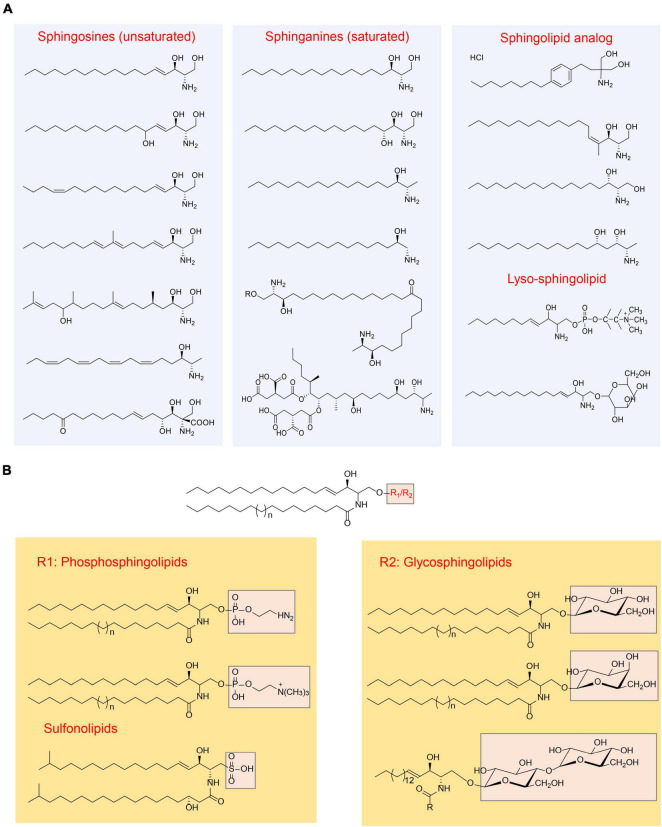
Overview of sphingolipids structure (Merrill, 2011). **(A)** Categorization of sphingolipid structures based on the “long chain” base; **(B)** categorization of sphingolipid structures based on the head group.

Sphingolipids are a group of lipids characterized by a common structural feature: they all consist of “long-chain” bases, also known as “sphingoid” bases, as the backbone, along with an amine group and two or three hydroxy groups at the structural end. The representative sphingoid base is sphingosine, specifically (2S, 3R, 4E)-2-aminooctadec-4-ene-1,3-diol, which is also referred as (E)-sphing-4-enine (Wieland Brown et al., 2013; Taniguchi and Okazaki, 2021). The sphingoid bases can vary in terms of the number and arrangement of hydroxyl groups, the length of alkyl chains, and the presence of saturated or unsaturated bonds (Merrill, 2011; Figure 1). Free sphingoid bases are present in minimal amounts, making them complex compounds. In case of complex sphingolipids, the amino group of the sphingoid bases undergoes acylation with fatty acids and/or a substituent at position 1 hydroxyl. The fatty acid chains can vary in length and number of double bonds, typically ranging from 14 to 36 carbon atoms and can be saturated, have a single double bond, or possess an α-hydroxyl group (Pruett et al., 2008). Common substituents include -H, fatty acids, phosphates, phosphocholine, phosphoethanolamine, galactose, glucose, sialic acid, sulfate, glucuronic acid, or a combination of these groups. Consequently, the potential diversity of lipid species is vast (Merrill et al., 2007). However, the actual number of species produced in biological systems is significantly lower, primarily due to the limited number of synthases involved in complex sphingolipid synthesis and their substrate specificities. In order to illustrate the sphingolipid structures clearly, the common structures were summarized in Figure 1.

Common sphingolipids include ceramide, sphingomyelin, glucosylceramide (GlcCer), lactosylceramide (LacCer), and galactosylceramides (GalCer). Additionally, there are more complex glycosphingolipids that contain a varying number of sugar residues. In addition to these, small amounts of sphingolipid analogs are present, along with “lyso-” sphingolipids. “Lyso-” sphingolipids refer to sphingoid bases combined with a headgroup but lacking the N-acyl substituent. Examples of “lyso-” sphingolipids include sphingosine 1-phosphate, sphingosine 1-phosphocholine, and lyso-glycosphingolipids (Figure 1). Moreover, there are *N*-methyl derivatives of sphingolipids and covalent adducts with proteins (Vesper et al., 1999; Merrill, 2011).

### 2.2. Bacterial sphingolipids

Initially, sphingolipids were believed to be present only in higher animals. However, further investigations have unveiled the presence of branched-chain sphingolipids in bacteria, including *Bacteroides*, *Prevotella*, *Sphingomonas*, *Sphingobacterium*, *Porphyromonas*, *Fusobacterium*, *Bdellovibrio*, *Cystobacter*, *Mycoplasma*, and *Flectobacillus*, which is noteworthy that a majority of these bacteria are anaerobes (Stoffel et al., 1975; Olsen and Jantzen, 2001).

In Stoffel et al. (1975) conducted an initial investigation on the lipid composition of anaerobic *B. thetaiotaomicron* metabolites. The analysis revealed that approximately 50% of the total lipid extract was sphingolipids, including sphingomyelin, ceramide phosphinicoethanolamine, free even-numbered and branched chain sphingosine bases and ceramide (Stoffel et al., 1975). More recently, in a germ-free mice mono-colonized with wild type *B. thetaiotaomicron* (BTWT) or *SPT* knocked *B. thetaiotaomicron* (BT_Δ_SPT), 144 unique bacterial lipids were identified using Tandem Mass Spectrometry, dependent on Spt, with 35 unique *Bacteroides* sphingolipids present in BTWT but absent in the BT_Δ_SPT. Among the differential sphingolipids, the most abundant ones in the BTWT-colonized mouse cecum were ceramide phosphoethanolamine (Cer-PE) and dihydroceramide (DHCer). Additionally, an abundant ceramide phosphoinositol (Cer-PI) was observed, a sphingolipid newly reported to be produced by gut *Bacteroides* strains and not detected in mammalian cells. Furthermore, a subset of phosphatidylethanolamine (PE) that were significantly more abundant in BT_Δ_SPT-colonized mouse cecum was discovered, with PE 32:0 and PE 35:07 being the most differential *in vivo* (Brown et al., 2019). Another study found that *B. thetaiotaomicron* can synthesize a set of uncharacterized homoserine-containing sphingolipids that are transferred to the host liver (Le et al., 2022).

Lipidomics analysis of the outer membrane vesicles (OMVs) from *B. thetaiotaomicron* VPI-5482 revealed the presence of diverse sphingolipids, glycerophospholipids, and glycine-serine dipeptide lipids (GS). The most abundant sphingolipids identified were ethanolamine phosphoceramide (EPC) and inositol phosphoceramide (IPC) (Sartorio et al., 2022). Another recent lipidomic analysis of four Bacteroides species reported that Dihydroceramide Phosphoethanolamine (DHCer-PE) is the most abundant sphingolipid across all four Bacteroides strains. Sphingolipids accounted for 19–29% of the total lipids detected in Bacteroides species, with DHCer making up approximately 20% of the sphingolipid fraction in *B. ovatus*, *B. vulgatus*, and *B. thetaiotaomicron*, but only 2% in *B. fragilis*. Instead, *B. fragilis* appeared to accumulate higher levels of keto-sphinganine (keto-sph). Additionally, detectable levels of sphinganine (sph) and deoxy-sph were found in both *B. thetaiotaomicron* and *B. ovatus*. α-Galactosylceramide (α-GalCer) was detected solely in *B. fragilis* and *B. vulgatus* (Ryan et al., 2023). Hence, there exists a diverse array of sphingolipid compounds, suggesting the potential existence of multiple pathways involved in sphingolipid biosynthesis across various *Bacteroides* species.

**FIGURE 2:**
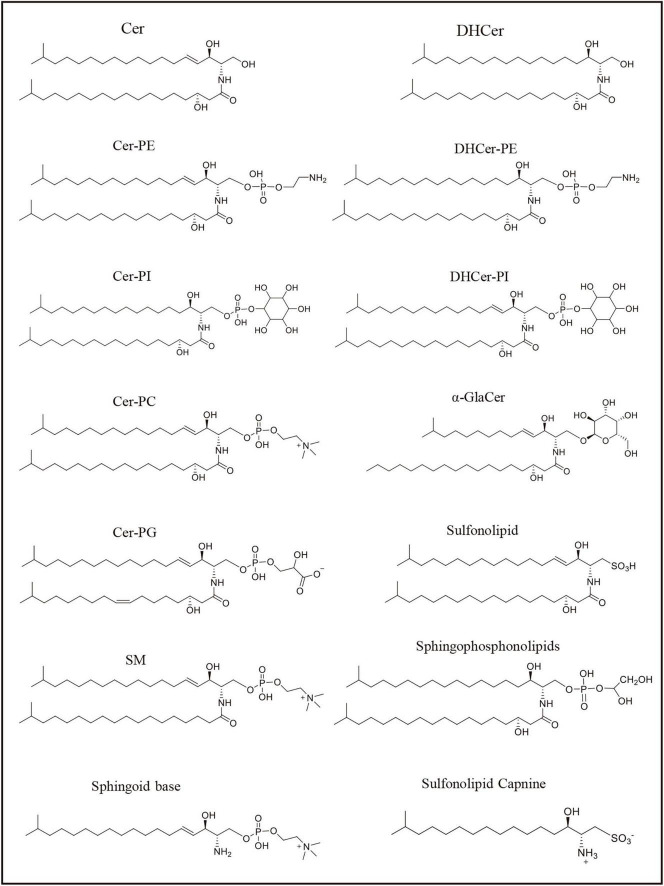
Sphingolipids represented structure mentioned in the Table 1. The formulas in the figure represent the representative structure, not the exact formulas.

In addition to the previous mentioned *Bacteroides*, a few members of the Chlorobi phylum, especially within genus *Chlorobium*, which are also capable of sphingolipid producing sphingolipids (Gupta and Lorenzini, 2007). Moreover, certain Alpha-Proteobacteria (such as *Acetobacter, Sphingomonas*, and *Novosphingobium*) and Delta-Proteobacteria (including *Myxococcus* and *Bdellovibrio*) are known to produce sphingolipids as well. While there are thousands of bacteria, only a small percentage of them are capable of producing sphingolipids (Table 1). Bacterial sphingolipids can be found in various environments, indicating the successful adaptation of sphingolipid producers in the biosphere (Fredrickson et al., 1995; Schubotz et al., 2009; Sollich et al., 2017; Heaver et al., 2018). For example, the human gastrointestinal tract is often heavily colonized by Bacteroidetes, including species like *Bacteroides* and *Prevotella* species, resulting to the existence of approximately 1 gram of sphingolipids produced by intestinal bacteria at any given time (Schnorr et al., 2014; Smits et al., 2017). Within the human gut, members of the Bacteroidetes phylum (e.g., *Bacteroides*, *Prevotella*, *Porphyromonas*) are known to synthesize sphingophospholipids that resemble sphingomyelin, a sphingolipid abundant in mammalian membranes. They also produce glycosphingolipids and DHCers (Stoffel et al., 1975; Olsen and Jantzen, 2001; Wieland Brown et al., 2013; Moye et al., 2016). Additionally, within the Bacteroidetes phylum, species such as *Chryseobacterium gleum*, *Alistipes*, and *Odoribacter* spp. were reported to synthesize sulfonolipids, as well (Walker et al., 2017; Heaver et al., 2018; Hou et al., 2022). Although the adaptation driving factors are unclear, it was reported that some pathogens, like *Bacillus cereus* (Flores-Díaz et al., 2016), *Clostridium perfringens* (Urbina et al., 2011), *Helicobacter pylori* (Chan et al., 2000), *Mycobacterium tuberculosis* (Speer et al., 2015), can survive in extreme environment because they produce sphingomyelinase, which degrade sphingomyelin (a kind of sphingolipids in cell membranes) and help pathogens invade into the host or escape from macrophage (Simonis and Schubert-Unkmeir, 2018). Moreover, certain opportunistic human pathogens, such as Sphingomonas species, are known to produce sphingolipids that likely originate from the plant rhizosphere (Berg et al., 2005). In the past years, increased human infections caused by opportunistic pathogens originating from the rhizosphere, which refers to the zone surrounding plant roots, have been noticed. It is worth noting that certain strains of Proteobacteria that are associated with plant roots can engage in interactions with both plants and humans. These particular strains have been found to possess beneficial effects on plant growth, displaying plant growth-promoting properties. Moreover, they have also shown excellent antagonistic properties against plant pathogens, offering potential benefits for both agricultural practices and the development of novel disease-control strategies (Berg et al., 2005; Bodenhausen et al., 2013; Erkosar and Leulier, 2014). This highlights the emerging significance of sphingolipid-producing *Proteobacteria* as important colonizers of both plants and animals. Examples include *Sphingomonas* spp. found on plant and root surfaces (Bodenhausen et al., 2013), as well as *Acetobacter* spp. associated with *Drosophila melanogaster* and *Caenorhabditis elegans* (Ogawa et al., 2010; Erkosar and Leulier, 2014; Zhang et al., 2017). The bacteria mentioned above are just a subset of the numerous species known to produce sphingolipids and more information are illustrated in Table 1 and Figure 2.

**TABLE 1:** Known bacterium producing sphingolipids.

Bacterium	Sphingolipids	References
*Acetobacter malorum*	Ceramide	Ogawa et al., 2010
*Alistipes finegoldii*	Sulfonolipids	Radka et al., 2022
*Algoriphagus machipongonensis*	Sulfonolipids	Alegado et al., 2012
*Alistipes and Odoribacter*	Sulfonolipids	Walker et al., 2017
*Bacteroides thetaiotaomicron*	Cer-PE, Cer-PG, Cer PI, Cer-PC, DHCer, DHcer-PE, DHcer-PI, sphingoid base,	Olsen and Jantzen, 2001; Le et al., 2022; Ryan et al., 2023
*Bacteroides fragilis*	Cer-PE, Cer-PG, DHCer, α-GalCer	Olsen and Jantzen, 2001; Ryan et al., 2023
*Bacteroides ovatus*	Cer-PE, Cer-PG, Cer-PI DHCer Sphingoid base,	Olsen and Jantzen, 2001; Ryan et al., 2023
*Bacteroides uniformis*	Cer-PE, Cer-PG	Olsen and Jantzen, 2001
*Bacteroides caccae*	Cer-PE, Cer-PG	Olsen and Jantzen, 2001
*Bacteroides eggerthii*	Cer-PE, Cer-PG	Olsen and Jantzen, 2001
*Bacteroides stercoris*	Cer-PE, Cer-PG	Olsen and Jantzen, 2001
*Bacteroides. vulgatus*	DHCer, Cer PE, Cer PI, α-GalCer	Ryan et al., 2023
*Bdellovibrio bacteriovorus*	Sphingophospholipids	Olsen and Jantzen, 2001
*Capnocytophaga ochracea*	Sulfonolipid Capnine	Liu et al., 2022
*Caulobacter crescentus*	Cer-PG	Dhakephalkar et al., 2023
*Chlamydia psittaci*	Sphingomyelin	Koch-Edelmann et al., 2017
*Chlamydia trachomatis*	Sphingomyelin	Koch-Edelmann et al., 2017
*Chryseobacterium gleum*	Sulfonolipids	Chaudhari et al., 2009; Hou et al., 2022
*Cystobacter fuscus*	Sphingolipids	Olsen and Jantzen, 2001
*Flectobacillus major*	Amino sphingophospholipids (namely Cer-PC)	Olsen and Jantzen, 2001
*Mycoplasma* spp.	Sphingophospholipid	Olsen and Jantzen, 2001
*Sphingobacterium* spp.	Sphingolipids	Olsen and Jantzen, 2001
*Porphyromonas gingivalis*	DHCer	Moye et al., 2016
*Prevotella melaninogenica*	Cer-PE, Cer-PG	Olsen and Jantzen, 2001
*Prevotella intermedia*	Cer-PE, Cer-PG	Olsen and Jantzen, 2001
*Prevotella bivia*	Cer-PE, Cer-PG	Olsen and Jantzen, 2001
*Porphyromonas gingivalis*	Cer-PE, Cer-PG	Olsen and Jantzen, 2001
*Sphingomonas* spp	Sphingolipids	Olsen and Jantzen, 2001
*Sphingomonas paucimobilis*	GlcCer, α-GalCe	Yard et al., 2007
*Sphingobacterium spiritivorum*	Ceramide	Minamino et al., 2003
*Salinibacter ruber*	Sulfonolipid	Corcelli et al., 2004

Cer-PE, ceramide phosphoethanolamine; Cer-PG, ceramide phosphoglycerol; Cer-PC, ceramide phosphocholine; Cer PI, ceramide phosphoinositol; DHCer, dihydroceramide; α-GalCer, α-Galactosylceramide; DHCer-PE, dihydroceramide phosphoethanolamine; DHCer-PI, dihydroceramide phosphoinositol, GlcCer, glucosylceramide.

### 2.3. The distinction between bacterial sphingolipids and eukaryotic sphingolipids

Recent research has uncovered that ability of both bacteria and eukaryotic organisms to produce sphingolipids (Olsen and Jantzen, 2001; Brown et al., 2019, 2023). However, there are notable distinctions between bacterial and eukaryotic sphingolipids. In mammals, the primary type of synthesized sphingolipids consists of even-chained linear structure. Conversely, bacterial sphingolipids exhibit a unique characteristic in which their sphingoid base has an odd chain length due to the presence of branched alkyl chains (Le et al., 2022; Ryan et al., 2023), like Threonine C_19_, Homoserine C_19_, and Serine/Alanine C_35_ DHCer, as well as Serine C_37_/C_35_ DHCer-PE in wild *B. thetaiotaomicron* (Le et al., 2022).

The initial step of sphingolipid synthesis in both bacteria and eukaryotes involves the condensation of an amino acid (typically serine in mammals) and a fatty acid (typically palmitate in mammals) through the action of the serine palmitoyltransferase (SPT) enzyme. This enzyme is highly conserved in both eukaryotes and bacteria (Yard et al., 2007). However, after the initial step, the pathways and products of sphingolipid biosynthesis diverge between eukaryotes and bacteria, including 3-Ketodihydrosphingosine reductase (KDSR), Sphingosine kinases (SKs), Sphingosine 1-phosphate lyase (S1PL) (Harrison et al., 2018; Heaver et al., 2018). Nevertheless, our understanding of bacterial sphingolipids is still less comprehensive compared to their eukaryotic counterparts, and therefore, the exact differences remain unclear. The specific variations in bacterial sphingolipid synthesis compared to eukaryotic sphingolipid synthesis have yet to be fully elucidated due to the relatively limited knowledge about bacterial sphingolipids. Further research is required to gain a more comprehensive understanding of bacterial sphingolipids and their distinctions from eukaryotic sphingolipids.

## 3. Interaction of bacterial sphingolipids with the host

**FIGURE 3:**
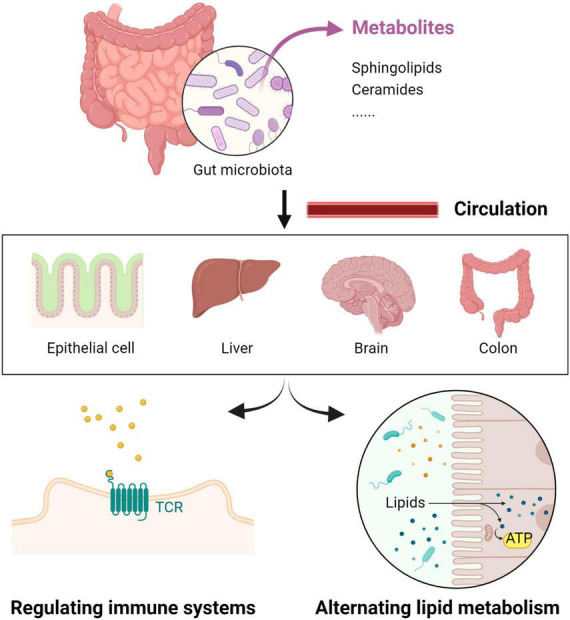
The potential link between host and microbial sphingolipids.

Efforts were undertaken to investigate the potential of bacterial sphingolipids to undergo transformation within the host organs. Fukami et al. (2010) conducted a study where ^13^C-labeled ceramide extracted from *Acetobacter malorum* were orally administered to mice. And these sphingolipids were readily absorbed and metabolized in the liver, ultimately forming complex sphingolipids (Fukami et al., 2010). Recent experiments utilizing fluorescently labeled bacteria demonstrated the presence of sphingolipids originating from bacteria in various tissues such as the liver, colon, ileum, brain, and skin, through the intestinal epithelial cells (Brown et al., 2019; Hussain et al., 2019; Johnson et al., 2020; Le et al., 2022). These findings suggest that bacterial sphingolipids play an active role in maintaining are actively utilized in maintaining sphingolipid homeostasis and promoting symbiosis within the host. Furthermore, these results provide evidence for a direct connection between host and microbial sphingolipids, as depicted in Figure 3.

### 3.1. Implications of bacterial sphingolipids on the immune system

The interaction between bacterial sphingolipids and the host began with the identification of a lipid produced by members of the Bacteroidetes that is analogous to alpha-GC, a known CD1-binding lipid derived from *Sphingomonas* species (Brown et al., 2023). Early studies revealed that alpha-GC derived from *Bacteroides fragilis* has the ability to bind to both mouse CD1d and the T-cell receptor (TCR) on invariant natural killer T (iNKT) cells, thereby inhibiting iNKT cell proliferation during neonatal development (Wieland Brown et al., 2013; An et al., 2014). Subsequent investigations have further elucidated the structural requirements for efficient binding between alpha-GC and CD1d, as well as the functional consequences of alpha-GC recognition by the TCR. These studies have highlighted the role of alpha-GC in regulating colonic NKT cells, suggesting its structure-specific immunomodulatory activity (Oh et al., 2021). Moreover, administration of these sphingolipids to mice has been found to induce an anti-inflammatory effect and reduce the number of colonic NKT cells (Oh et al., 2021).

Further support for the impact of microbiome sphingolipids on host inflammatory and metabolic pathways was obtained through experiments conducted on germ-free mice colonized with sphingolipid-deficient bacteria. These mice exhibited gut inflammation and alterations in host ceramide levels (Brown et al., 2019). The interaction of *Bacteroides* sphingolipids with the innate immune system was also observed, as sphingolipids present in the outer membrane of *Bacteroides* facilitated a tolerant immune response. Studies on Bacteroidetes demonstrated that sphingolipids in outer membrane vesicles (OMVs) acted as agonists for TLR2 signaling in macrophages, thus playing a critical role in limiting inflammatory signaling (Rocha et al., 2021). Analysis of an inflammatory bowel disease (IBD) metabolomic dataset indicated reduced abundance of *Bacteroides* sphingolipids in IBD cases, along with negative correlations with inflammation and host sphingolipid production (Brown et al., 2019). These findings, coupled with the observation that bacterial sphingolipids are capable of translocating into the host (Yatsunenko et al., 2012), highlight the significance of bacterial sphingolipids in maintaining immune homeostasis.

### 3.2. Influence of bacterial sphingolipids on host metabolites

A recent study demonstrated that exposure of mice to *B. thetaiotaomicron*, a producer of sphingolipids, resulted in a reduction in the *de novo* production of sphingolipids by the host, while liver ceramide levels increased. Furthermore, experiments conducted on human cell cultures and mouse models confirmed that *Bacteroides* sphinganine can be taken up by host epithelial cells and incorporated into sphingolipid metabolic pathways. These findings suggest that gut-derived sphingolipids have an impact on host lipid metabolism and liver function (Johnson et al., 2020). Further investigation into the relationship between the gut and liver regarding sphingolipid signaling unveiled that the mere presence of microbiome sphingolipids was sufficient to reverse fatty liver disease in mice (Le et al., 2022). This indicates that microbiome sphingolipids not only exert local effects within the gut but also possess systemic effects by trafficking to organs beyond the intestine, thereby modulating host sphingolipid signaling.

## 4. Microorganisms and dietary sphingolipids

Sphingolipids are essentially widespread components found in various foods, especially in eggs and milk, that we consume on a daily basis (Andrieu-Abadie and Levade, 2002; Hussain et al., 2019), While it is believed that most sphingolipids, including sphingomyelin (SM), are not absorbed intact in the upper intestine, they do enter the distal small intestine, where they undergo degradation and give rise to bioactive lipids such as ceramide and sphingosine (Duan et al., 2007; Fischbeck et al., 2009; Norris et al., 2019). Research studies have indicated the presence of approximately 10% undegraded sphingomyelin and 30–90% ceramide in mouse feces (Norris et al., 2019). Furthermore, analysis of fecal metabolome of urban-dwelling Italians and the Hadza people of Tanzania revealed an overall abundance of sphingolipids (Turroni et al., 2016).

A study discovered that the addition of gangliosides, a type of sphingolipid, to infant formula had a significant impact on the intestinal ecosystem of preterm newborns. This supplementation led to an increase in the presence of *Bifidobacteria* and a decrease in the abundance of *Escherichia coli* (Rueda, 2007). *Bacteroidetes*, including *Bifidobacterium* strains, possess enzymes that can break down gangliosides, such as sialidases that release free sialic acid. This biochemical process contributes to the immunological functions and prevention of infections (Rueda, 2007; Lewis and Lewis, 2012). Interestingly, *Bifidobacterium* strains, despite lacking the ability to synthesize sphingolipids, can still import and utilize sphingolipids to generate DHCer (Lee et al., 2021). On the other hand, the pathogenic bacterium *Clostridium perfringens*, which can be found in stool samples, produces an enzyme called sphingomyelinase. This enzyme hydrolyzes sphingomyelin into ceramide and phosphocholine (Urbina et al., 2011; Wang et al., 2021).

In more recent findings, conclusive evidence of the microbial uptake of dietary sphinganine in the mouse gut has been established using a click-chemistry based method to trace the incorporation of bio-orthogonal dietary omega-alkynyl sphinganine into the gut microbial community. The study identified several bacterial genera, including *Bacteroides*, *Prevotella*, *Bifidobacterium*, *Lactobacillus*, and *Turicibacter*, as participants in the assimilation process of sphinganine. Of particular interest, over 99% of the bacteria involved in the assimilation were identified as *Bacteroides*, with *Prevotella* being the second most prevalent, albeit in much lesser abundance compared to *Bacteroides* (Lee et al., 2021). Based on these findings, it can be inferred that known sphingolipid-producing bacteria, primarily *Bacteroides*, play a dominant role in the metabolism of dietary sphinganine.

## 5. Conclusion

Among sphingolipids characterized by long-chain bases with an amine group and hydroxy groups at the structural end, bacterial sphingolipids consist of odd chain lengths due to the presence of branched alkyl chains, unlike mammalian sphingolipids. The interaction between bacterial sphingolipids and the host is a complex and dynamic process, although our understanding of it is limited. Current knowledge suggests that bacterial sphingolipids have the ability to translocate from epithelial cells to body organs, thereby impacting the immune system and metabolism of the host. Furthermore, undegraded dietary sphingolipids in the distal small intestine are exposed to the gut microbiome, which can break them down into bioactive lipids. These findings emphasize the intricate interplay of sphingolipids within the host, underscoring the importance of bacterial sphingolipids in maintaining inner homeostasis and overall health.

However, our understanding of microbial sphingolipids in the gut is still limited, and further research is needed to fully explore their potential. Several key areas warrant investigation. Firstly, it is important to identify the specific bacteria responsible for sphingolipid production and characterize the types of sphingolipids they produce. Understanding the translocation mechanisms of these sphingolipids and their transformation processes is also essential. Additionally, investigating the functions of bacterial sphingolipids within the host, including their impact on the immune system and metabolic homeostasis, is crucial. Since the gut microbiome functions as a vital organ in the host, conducting more comprehensive studies in this field will provide valuable insights that can potentially lead to the development of therapeutics targeting sphingolipid metabolism and improving the various diseases. Advancements in these areas could have profound implications for human health and overall wellbeing.

## Author contributions

XB: Writing – original draft. RY: Writing – review and editing. XT: Writing – review and editing. MC: Writing – review and editing.
